# A Multimodal System for Comprehensive Cardiovascular Monitoring Using ECG, PCG, and PPG Signal Fusion

**DOI:** 10.3390/s25216708

**Published:** 2025-11-02

**Authors:** Khang Thanh Tran, Thao Nguyen Tran, Dang Nguyen Huynh, Nguyen Khoa Le, Cao Dang Le, Huu Xuan Mai, Quang Linh Huynh, Trung Hau Nguyen

**Affiliations:** 1 Department of Biomedical Engineering, Faculty of Applied Science, Ho Chi Minh City University of Technology (HCMUT), 268 Ly Thuong Kiet Street, Dien Hong Ward, Ho Chi Minh City 700000, Vietnam; 2 Vietnam National University Ho Chi Minh City, Linh Xuan Ward, Ho Chi Minh City 700000, Vietnam

**Keywords:** cardiovascular system, electrocardiogram (ECG), phonocardiogram (PCG), photoplethysmogram (PPG), data fusion

## Abstract

This study proposes a low-cost, wearable multimodal system for comprehensive cardiovascular monitoring, integrating electrocardiogram (ECG), phonocardiogram (PCG), and photoplethysmogram (PPG) signals. By leveraging the complementary strengths of these modalities, the system extracts key physiological markers: pre-ejection period (PEP) from ECG–PCG fusion to assess myocardial electromechanical timing; pulse transit time (PTT) from PCG–PPG fusion to reflect arterial stiffness; and pulse arrival time (PAT) from ECG–PPG fusion to characterize heart–arterial coupling. Experimental results show that PEP is significantly associated with body mass index (BMI) but not age, suggesting that age-related changes primarily affect the arterial system rather than myocardial function. In contrast, both PTT and PAT demonstrate moderate negative correlations with age and BMI, supporting their relevance as noninvasive indicators of vascular aging. Additionally, PAT exhibits a significant sex-based difference, highlighting physiological disparities between male and female cardiovascular systems. Overall, the proposed fusion-based approach demonstrates technical feasibility and clinical potential for scalable, preventive cardiovascular healthcare, enabling early risk detection, continuous at-home monitoring, and improved long-term health management.

## 1. Introduction

Cardiovascular diseases (CVDs) remain a leading cause of global mortality and morbidity, highlighting the critical need for early detection and continuous monitoring to support preventive healthcare. Conventional cardiovascular assessment tools such as echocardiography, arterial tonometry, and catheter-based measurements, often require complex, costly setups, limiting their feasibility for routine use in large-scale screening or home-based monitoring [1,2,3]. These limitations emphasize the demand for affordable, non-invasive, and scalable monitoring solutions capable of reliably capturing key cardiovascular markers for widespread preventive care.

Recent advancements in sensor technology have facilitated the simultaneous acquisition and fusion of multiple physiological signals such as phonocardiogram (PCG), electrocardiogram (ECG), and photoplethysmogram (PPG) [4,5]. Each signal provides unique yet complementary information: ECG reflects the heart’s electrical depolarization and repolarization, PCG captures mechanical heart sounds linked to valve motion and ventricular activity, and PPG measures pulsatile blood volume changes indicative of peripheral vascular dynamics. While these modalities individually offer valuable insights into cardiovascular status, their integration significantly enhances diagnostic capability by jointly characterizing cardiac electromechanical function and arterial pulse propagation, thereby enabling a more comprehensive assessment of cardiovascular health [6,7,8].

Among the temporal biomarkers derived from these signals, pulse transit time (PTT), the interval required for the arterial pulse wave to travel between two measurement sites, is a widely studied surrogate of arterial stiffness and vascular compliance [9,10,11]. PTT has been extensively investigated as a non-invasive indicator of hypertension, vascular aging, and overall cardiovascular risk. Prior studies consistently demonstrate that age is a major determinant of arterial stiffness, with PTT typically decreasing as arterial walls lose elasticity over time [12,13,14]. Although factors such as body mass index (BMI) have been explored in relation to arterial stiffness, their predictive value remains uncertain [15,16,17].

Beyond PTT, integrating ECG, PCG, and PPG enables the extraction of additional diagnostic features. The pre-ejection period (PEP), measured as the interval between the ECG R-peak and the first heart sound (S1) in the PCG, reflects ventricular contractility and autonomic regulation, offering insights into cardiac workload and sympathetic activity. When combined with PTT, the pulse arrival time (PAT), the total interval from the ECG electrical trigger to the peripheral PPG waveform, serves as a comprehensive marker encompassing both myocardial electromechanical function and arterial dynamics. Previous studies have reported that PAT may exhibit sex-specific differences in cardiovascular response, potentially influenced by variations in vascular tone, hormonal factors, and cardiac structure [8,11].

Building on these insights, this study introduces a novel, low-cost multimodal system that concurrently acquires ECG, PCG, and PPG signals to extract PEP, PTT, and PAT for comprehensive cardiovascular assessment. Unlike prior studies that focus on dual-signal combinations (e.g., ECG–PPG or ECG–PCG) [6,18], our approach integrates all three modalities within a unified, wireless, and open-source ESP32-based platform, capturing electrical, mechanical, and vascular dynamics simultaneously. By examining associations with age, BMI, and sex, the system aims to identify non-invasive biomarkers of vascular aging and cardiac stress. Its scalable, cost-effective design supports early detection, continuous monitoring, and broader application in preventive healthcare.

## 2. Methods

### 2.1. The Proposed Vital-Sign Monitoring System

#### 2.1.1. Simplified Circuitry

**Figure 1:**
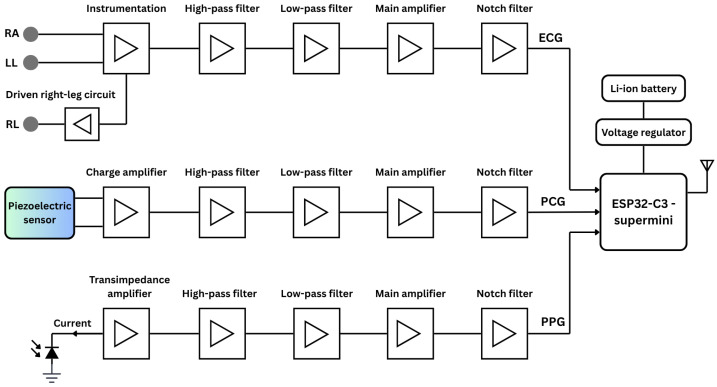
The simplified functional block diagram of the proposed vital-sign monitoring system comprises the analog circuits for ECG, PCG, and PPG acquisition, as well as the digital components, including the microcontroller and power supply.

Figure 1 presents an overall functional block diagram of the proposed system, including simplified analog circuits for ECG, PCG, and PPG signal acquisition. The system is powered by lithium-ion polymer (Li-Po) batteries, with a buck/boost converter providing stable DC voltage. At the core of the system is the ESP32 microcontroller (Espressif Systems, Shanghai, China), specifically the ESP32-C3 Supermini board-a compact, 160 MHz development board featuring integrated Wi-Fi and Bluetooth communication protocols.

##### ECG Circuitry

Three dedicated conditioning circuits are implemented for ECG, PPG, and PCG measurements. The simplified ECG circuit amplifies the voltage difference between two Ag/AgCl electrodes, known for low noise and impedance. The signal passes through an EMI filter before reaching an INA128 instrumentation amplifier (Texas Instruments, Dallas, TX, USA), chosen for its low-voltage operation and high noise rejection. A 0.5 Hz second-order high-pass filter removes baseline drift, followed by a 49 Hz fourth-order low-pass filter to suppress high-frequency noise [19]. The output is then amplified to match the ADC’s 0–5 V range, and a final 50 Hz notch filter eliminates residual power-line interference.

##### PCG Circuitry

PCG signals were captured using a piezoelectric sensor (STD1-028K PZ, TE Connectivity, Galway, Ireland) with a wide and flat frequency response extending up to 10 MHz. The piezoelectric sensor output is first routed through a charge amplifier with a gain of 1.25. The signal then passes through a second-order high-pass filter (20 Hz cutoff) to eliminate low-frequency drift, followed by a fourth-order low-pass filter (500 Hz cutoff) to attenuate high-frequency noise [20]. Further amplification is provided by an INA128 instrumentation amplifier, and a second-order notch filter is applied to suppress 50 Hz power-line interference. Prior to ADC conversion by the microcontroller, the conditioned signal is biased to the appropriate level.

##### PPG Circuitry

For PPG acquisition, a BPW-34 photodiode (Malvern, PA, USA) paired with a 950 nm near-infrared LED was used in a finger clip to ensure stable placement and reduce motion and ambient light interference. The photocurrent is converted to voltage via a transimpedance amplifier, then filtered by a 0.07 Hz high-pass filter to remove DC offset. A non-inverting amplifier and 5 Hz fourth-order low-pass filter suppress high-frequency noise [21]. The signal is further conditioned by an inverting amplifier, another low-pass filter, and a 50 Hz notch filter for anti-aliasing and baseline biasing to match the ADC range while preserving waveform fidelity.

All active stages of the analog circuit, encompassing amplification and filtering, utilize LM324 operational amplifiers (Texas Instruments, Dallas, TX, USA) selected for their low input offset voltage, minimal bias current, and strong EMI immunity. The three signals are fed into separate channels of a shared ADC on the microcontroller, where they are digitized and subsequently transmitted wirelessly to a personal computer for further digital signal processing.

#### 2.1.2. Photograph of the Proposed System Prototype

**Figure 2:**
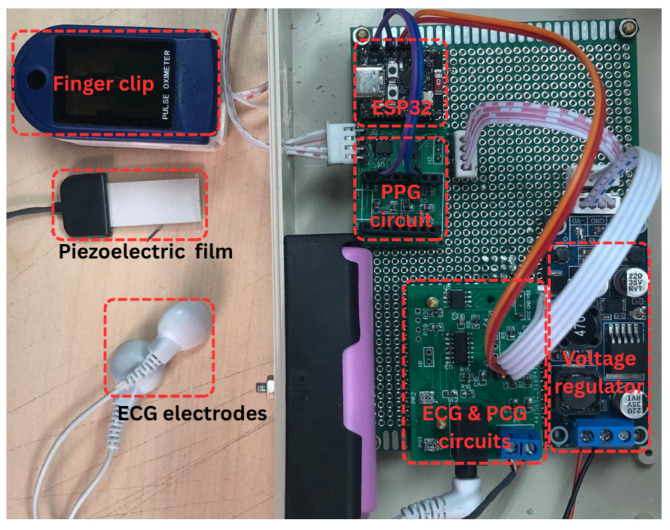
Photograph of the proposed system.

Figure 2 presents a photograph of the complete system and its components. The left portion of the image displays the PPG finger clip, piezoelectric sensor, and ECG electrode clip, used for PPG, PCG, and ECG signal acquisition, respectively. The right portion illustrates the circuitry, comprising the PPG, PCG, and ECG analog front-end circuits, the ESP32-C3 Supermini microcontroller, and the power supply.

### 2.2. Experimental Protocol

**Figure 3:**
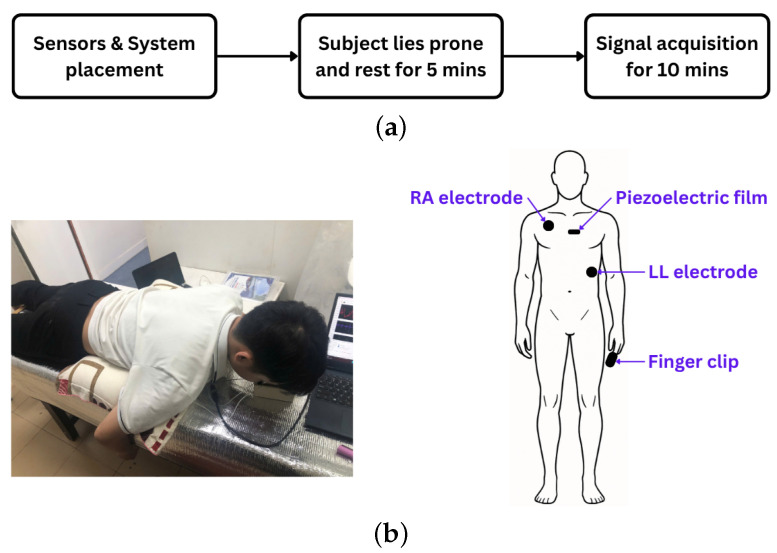
(**a**) Experimental protocol. (**b**) Experimental setup (**left**) and sensor placement on the human body (**right**).

The experiment commenced with the placement of ECG electrodes, a piezoelectric sensor, and a PPG finger clip on each participant. ECG electrodes were positioned at the right arm (RA) and left leg (LL) sites, while the piezoelectric sensor was affixed at the P3 location, centrally along the sternum. For PPG acquisition, participants inserted their index finger into the finger clip during the experiment (Figure 3a).

Fourteen healthy volunteers (8 males, 6 females; aged 20–44 years) participated. Body weight and height were recorded, and written informed consent was obtained. To ensure baseline physiological states, participants abstained from coffee and smoking for at least two hours before testing. All had non-elevated blood pressure, defined by the 2024 ESC guidelines as office systolic <120 mmHg and diastolic <70 mmHg (with equivalent home and ambulatory thresholds) [22].

Following sensor placement, participants rested in a prone position for five minutes to allow stabilization of physiological signals. Data acquisition commenced after stabilization and continued for ten minutes (Figure 3b). The recorded signals were stored for subsequent analysis.

### 2.3. Signal Processing

**Figure 4:**
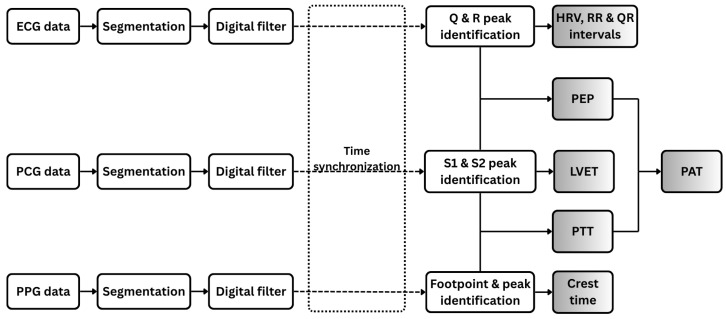
Workflow of digital signal processing for ECG, PCG, and PPG data. Each signal type is segmented and filtered prior to feature extraction. Extracted features include both individual signal-based features and combined features derived from pairs of signals.

Figure 4 illustrates the signal processing workflow applied to the three physiological signals analyzed in this study: PCG, ECG, and PPG.

#### 2.3.1. ECG Signal Processing and Feature Extraction

The ECG signal is segmented into individual cardiac cycles and processed using a digital bandpass filter (0.5–49 Hz) to preserve essential cardiac electrophysiological components while attenuating baseline wander. A 50 Hz notch filter is subsequently applied to suppress power-line interference. A peak detection algorithm [23] is employed to accurately identify the Q, R, and S peaks of the ECG waveform, delineating ventricular depolarization events. From these detected fiducial points, the R–R and Q–R intervals, along with heart rate variability (HRV) metrics, are computed as diagnostic features.

#### 2.3.2. PCG Signal Processing and Feature Extraction

In parallel, the PCG signal is segmented into individual cardiac cycles. Each segment is bandpass filtered (20–500 Hz) to retain cardiac-specific frequency components and processed with a 50-Hz notch filter to suppress power-line interference. Wavelet decomposition using the Daubechies-7 (db7) wavelet is then applied to enhance prominent features while attenuating background noise [24]. During each cardiac cycle, the first heart sound (S1), signaling the closure of the mitral and tricuspid valves at the onset of ventricular systole, and the second heart sound (S2), marking the closure of the aortic and pulmonary valves at the end of systolic ejection, are precisely detected as key temporal landmarks. The left ventricular ejection time (LVET) is subsequently calculated as the interval between S1 and S2, normalized by heart rate to account for inter-individual variability [25].

#### 2.3.3. PPG Signal Processing and Feature Extraction

For the photoplethysmogram (PPG) signal, individual pulse waveforms are segmented and subsequently filtered using a narrow bandpass filter (0.1–5 Hz) to preserve cardiac-related pulsatile components while attenuating noise. Within each filtered waveform, the footpoint (onset of the pulse) and systolic peak are identified, and the interval between these points, referred to as the crest time is measured. Crest time quantifies the duration required for the peripheral blood volume to reach its maximum following the initial systolic upstroke and serves as a sensitive marker of arterial stiffness and vascular resistance [26]. Additionally, the intervals between consecutive systolic peaks are computed to assess pulse rhythm variability.

#### 2.3.4. Fused Feature Extraction

All signals (ECG, PCG, and PPG) were acquired simultaneously using a unified data acquisition system based on the ESP32-C3 microcontroller, which employed a shared 12-bit ADC sampling at 1 kHz to ensure synchronized collection. Channel latency was experimentally validated to be under 2 ms by applying identical test inputs across multiple ADC channels and measuring inter-channel delays. This latency is minimal compared to physiological intervals of interest, such as PEP and PTT, which typically range from tens to hundreds of milliseconds. After preprocessing, temporal alignment was refined by compensating for processing delays introduced by the microcontroller, ensuring accurate synchronization of cardiac events across modalities (Figure 4).

From the synchronized signals, key temporal features are extracted, including PEP and PTT. PEP, a marker of cardiac contractility and autonomic function, is measured as the interval between the R-peak of the ECG and the corresponding S1 peak in the PCG, representing the delay between ventricular depolarization and the onset of blood ejection [27,28]. PTT, reflecting the travel time of the arterial pulse wave from the heart to the periphery, is calculated as the interval from the S1 peak in the PCG to the footpoint of the corresponding PPG waveform, representing the time for the pressure wave to reach a distal site, such as the finger [29,30]. PAT, capturing the combined effects of cardiac electromechanical activation, arterial compliance, and peripheral resistance, is derived as the sum of PEP and PTT [31,32]. Collectively, these features provide a comprehensive characterization of cardiovascular dynamics, enabling detailed physiological assessment.

Pearson correlation coefficients were used to assess linear relationships across all regression analyses in this study, with corresponding *p*-values and 95% confidence intervals reported to evaluate statistical significance.

## 3. Results and Discussions

### 3.1. Data Visualization

**Figure 5:**
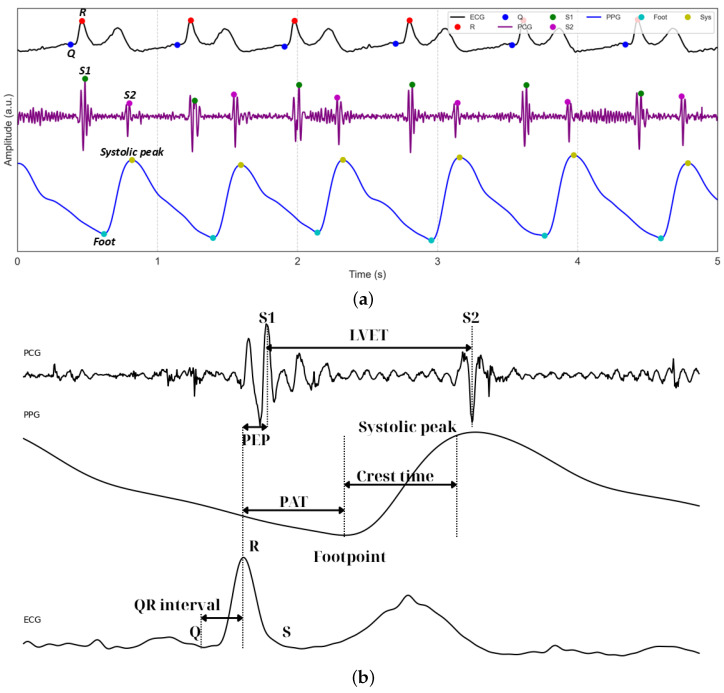
(**a**) Example of segmented and filtered ECG, PCG, and PPG signals stacked together, with the peak detection algorithm applied. (**b**) Example of how PEP, PAT, and PTT are related.

Figure 5a displays representative 5 s waveforms of ECG, PCG, and PPG signals acquired using the proposed system. The figure highlights the performance of the peak detection algorithms, which accurately identify the Q and R peaks within the QRS complexes of the ECG. For the PCG waveform, the first (S1) and second (S2) heart sounds are clearly detected, serving as critical temporal landmarks for cardiac cycle analysis. In the PPG waveform, the footpoints corresponding to the onset of pulsatile blood flow are precisely identified. Figure 5b illustrates the subsequent extraction of key temporal features, including LVET and crest time, as well as composite metrics such as PEP, derived from ECG and PCG, and PAT, integrating ECG and PPG.

**Figure 6:**
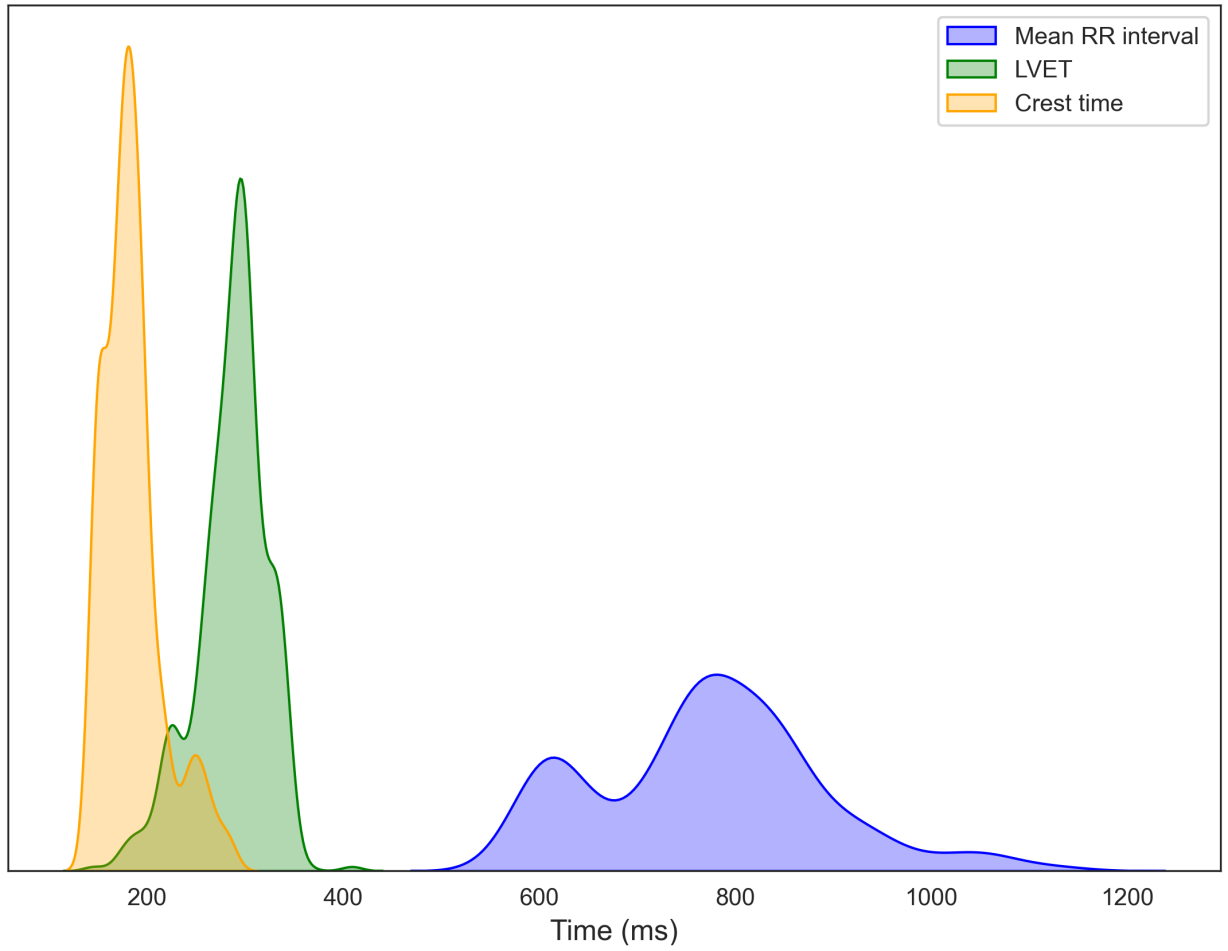
Distributions of R–R interval (RRI) from ECG, left ventricular ejection time (LVET) from PCG, and crest time from PPG, computed from segmented recordings and aggregated across all participants.

Figure 6 depicts the distributions of RRI, LVET, and crest time, extracted from 15-s segmented windows of ECG, PCG, and PPG signals collected from 14 participants. Each histogram is overlaid with a kernel density estimation (KDE) curve to better illustrate overall data trends. The RRI distribution exhibits a primary cluster between 750 and 850 ms, with considerable inter-individual variability, consistent with normative adult values [33,34]. A secondary peak at shorter intervals suggests population heterogeneity, potentially distinguishing individuals at rest from those experiencing stress or autonomic imbalance [35,36]. LVET, representing the duration of left ventricular ejection and a key marker of systolic function, predominantly ranges from 250 to 350 ms, aligning with established healthy adult values [37]. For crest time, derived from the PPG signal, most participants exhibit values between 175 and 190 ms, typical of healthy vascular dynamics [21]. The extended right tail toward longer crest times may indicate a subset of individuals with increased arterial stiffness or elevated vascular resistance [38,39]. Further data exploration, including regression modeling and statistical analyses, is presented in the following sections.

### 3.2. BMI and Age Associations with Vital Signs

#### 3.2.1. Relationships of BMI and Age with Primary Cardiovascular Features: RRI, LVET, and Crest Time

**Figure 7:**
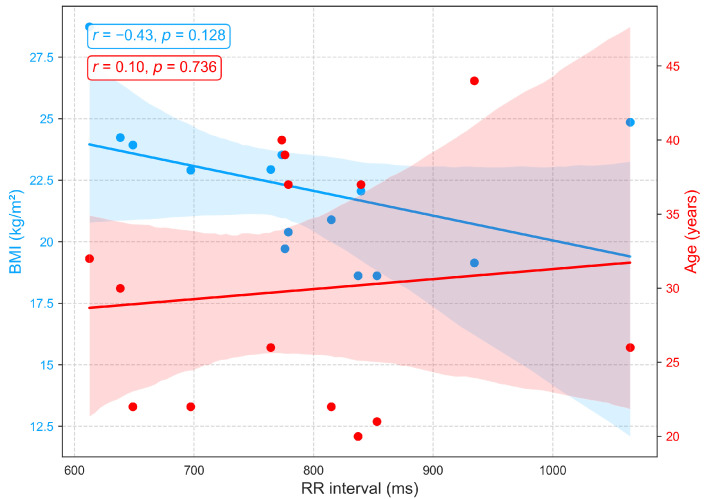
Correlation of RR interval, an ECG-derived heart rate variability metric, with age and BMI.

Figure 7 shows that the RR interval (RRI), derived from ECG recordings, exhibits a weak positive correlation with age (r=0.1) among individuals aged 22 to 44, indicating a slight trend toward longer RRI and consequently lower resting heart rates, a pattern consistent with previous findings [40]. The small effect size suggests that additional factors, including physical fitness, metabolic status, circadian rhythms, and psychological or environmental stressors, contribute substantially to inter-individual variability. Conversely, a moderate negative correlation is observed between RRI and BMI (r=−0.43), with higher BMI associated with shorter RRI and elevated resting heart rates. These findings suggest that BMI exerts a stronger influence than age on autonomic balance and resting heart rate, consistent with prior reports documenting significant RRI reductions across BMI categories [40,41].

**Figure 8:**
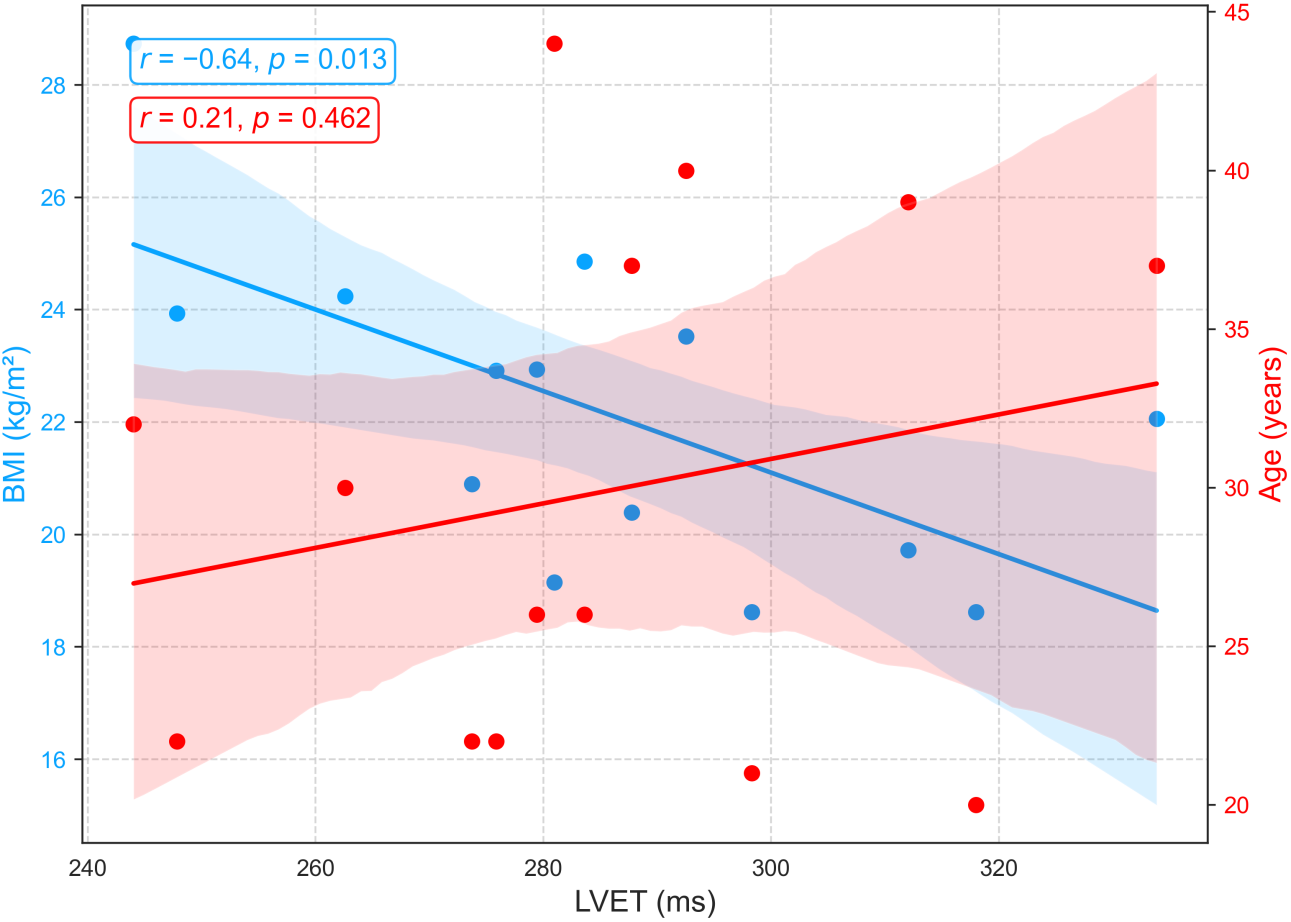
Correlation of LVET, a PCG-derived index of systolic function, with age and BMI.

Figure 8 depicts the associations between LVET, derived from PCG, and demographic variables. A weak positive correlation with age (r=0.21) indicates a slight tendency for LVET to increase with advancing age, possibly reflecting age-related prolongation of systolic ejection due to reduced ventricular compliance and contractile efficiency. However, the low correlation suggests that age has only a minor influence on LVET within this cohort, consistent with previous findings of a gradual age-related increase [42,43]. In contrast, a moderate negative correlation is observed between LVET and BMI (r=−0.64), with longer LVET generally associated with lower BMI. This relationship may reflect hemodynamic characteristics in leaner individuals, such as reduced afterload and more efficient ventricular ejection, whereas elevated BMI is often linked to arterial stiffness, increased afterload, and compensatory shortening of ejection time. Although prior studies have not explicitly reported a direct LVET-BMI relationship, excess body weight has been associated with impaired systolic function, including reduced ejection fraction and abnormal systolic time intervals [44,45].

**Figure 9:**
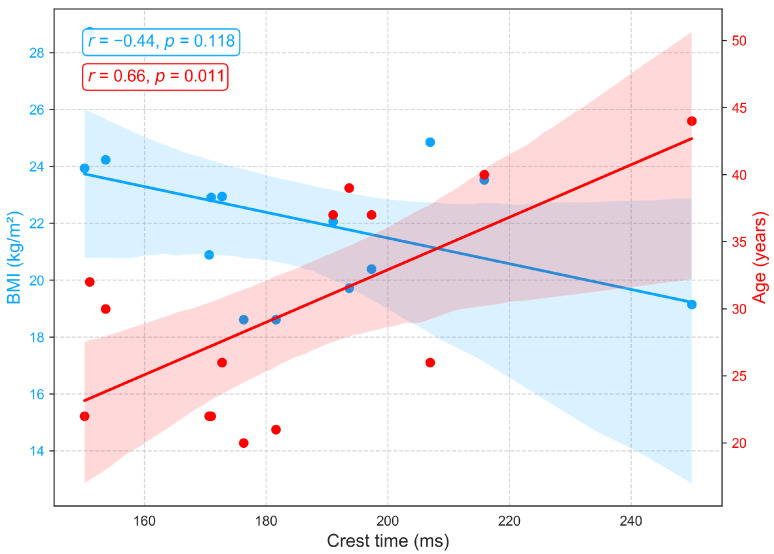
Correlation of crest time, a PPG-derived measure of arterial compliance and pulse dynamics, with age and BMI.

Figure 9 shows the associations between crest time (a PPG derived indicator of arterial compliance and pulse dynamics) and demographic variables. A strong positive correlation with age (r=0.66, p<0.05) demonstrates that crest time increases with advancing age, likely reflecting arterial stiffening, diminished vascular elasticity, and slower systolic–diastolic transitions that delay the systolic peak [46,47]. In contrast, a moderate negative correlation with BMI (r=−0.44) suggests that longer crest times are more prevalent among individuals with lower BMI, potentially indicative of greater arterial compliance and reduced peripheral resistance, resulting in slower, smoother pulse upstrokes. Higher BMI, often linked to arterial stiffness, elevated resistance, and accelerated systolic rise, corresponds to shorter crest times. As no prior studies have directly established this relationship between crest time and BMI, this finding warrants further investigation.

#### 3.2.2. Relationships of BMI and Age with PEP

**Figure 10:**
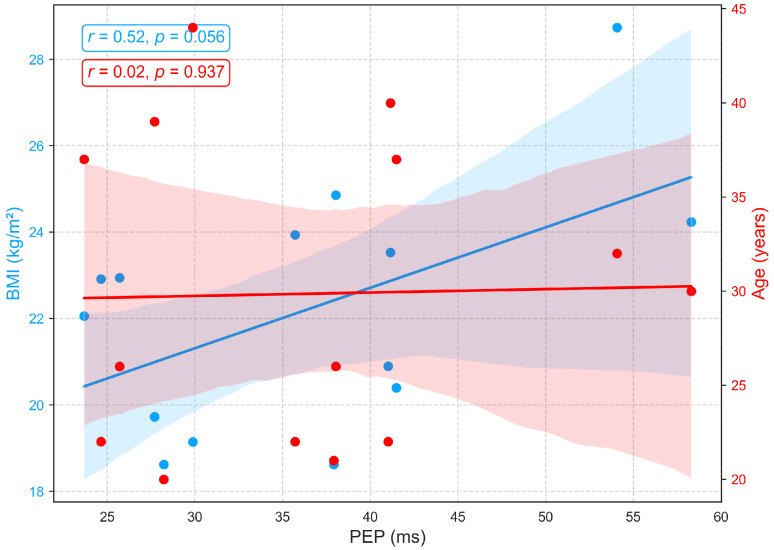
Relationships between PEP with age and BMI.

Figure 10 illustrates the relationships between age, BMI, and PEP, a measure of electromechanical coupling between cardiac electrical activation (ECG) and mechanical contraction. PEP shows no significant association with age (r=0.02, p=0.937), aligning with prior studies that report its relative stability across age groups in the absence of cardiac pathology [48,49]. Conversely, a positive correlation (r=0.52, p=0.056) between BMI and pre-ejection period (PEP) suggests that individuals with higher body mass index may face increased cardiac afterload, meaning the left ventricle encounters greater resistance when ejecting blood. This added load extends the isovolumetric contraction phase, resulting in a longer PEP. In practical terms, an increased PEP reflects a greater effort by the myocardium to build sufficient pressure to open the aortic valve. This pattern may indicate early subclinical cardiac strain or mild contractile dysfunction, commonly associated with obesity and elevated sympathetic tone [44,50].

#### 3.2.3. Relationships of BMI and Age with PTT

**Figure 11:**
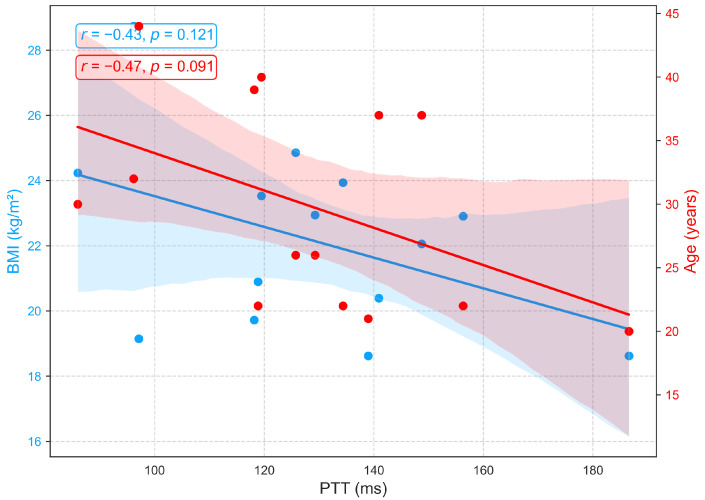
Relationships between PTT with age and BMI.

Figure 11 demonstrates that PTT, an indicator of arterial stiffness, exhibits only weak associations with BMI and age in this cohort. Moderate negative correlations with age (r=−0.47, p=0.09) and BMI (r=−0.44, p=0.12) were observed but did not reach statistical significance. The trend toward shorter PTT with advancing age is consistent with vascular aging, which is characterized by arterial stiffening, reduced elasticity, and increased pulse wave velocity, yet the nonsignificant results suggest that these effects are modest in this sample. Additionally, recent studies report that PTT shows minimal sensitivity to body composition, with no consistent association between BMI and PTT [51,52].

#### 3.2.4. Relationships of BMI and Age with PAT

**Figure 12:**
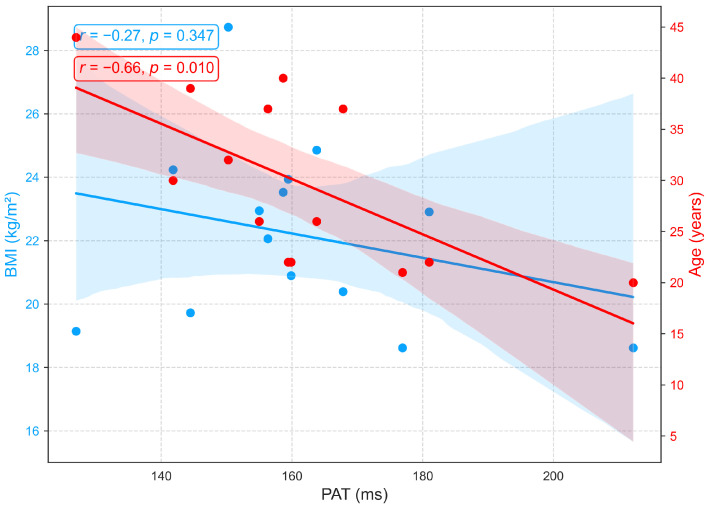
Relationships between PAT with age and BMI.

Although PAT is often used interchangeably with PTT for blood pressure estimation [53], PAT differs because it extends from the ECG R wave to the PPG peak and therefore includes the preejection period, the interval from the ECG R wave to the opening of the aortic valve [31]. In this study, PEP, PTT, and PAT were analyzed as distinct parameters. Figure 12 shows that PAT is strongly influenced by age but minimally by BMI. A significant negative correlation with age (r=−0.66, p<0.05) reflects vascular aging, which is characterized by arterial stiffening, reduced compliance, and increased pulse wave velocity, changes that accelerate wave propagation and shorten PAT. In contrast, the weak, non-significant correlation with BMI (r=−0.27, p=0.347) suggests that body composition exerts little direct effect on PAT in this cohort. Overall, PAT primarily reflects arterial changes related to age rather than hemodynamic factors associated with BMI, an aspect seldom addressed in prior research. Similar to PTT, PAT exhibits negative correlations with both age and BMI (Figure 11 and Figure 12), highlighting their close physiological relationship and conceptual overlap [53].

#### 3.2.5. Influence of Sex on Cardiovascular Function

**Figure 13:**
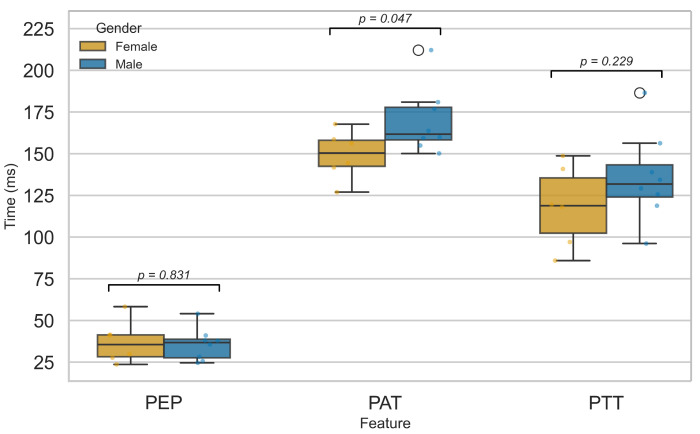
Comparison of PEP, PTT, and PAT features between male and female groups.

Figure 13 compares PEP, PTT, and PAT between male and female participants. No significant sex differences were found for PEP or PTT, suggesting that myocardial electromechanical function and arterial pulse transmission are not markedly affected by sex, in agreement with prior studies [52,54]. In contrast, PAT showed a significant difference (p<0.05), likely reflecting physiological variations in cardiovascular structure and function between sexes. Females typically exhibit higher resting heart rates, smaller arterial diameters, and distinct vascular tone regulation, all of which may influence the delay between cardiac depolarization and peripheral pulse arrival [55,56,57,58,59]. These factors likely contribute to the observed PAT difference, even in the absence of significant changes in PEP or PTT. This finding is consistent with reports by Dehghanojamahalleh et al. [60], who observed sex-based differences in finger-based PAT and PTT but not in toe-based measurements.

## 4. Conclusions

This study presents a novel, cost-effective multimodal system for cardiovascular health monitoring, integrating ECG, PCG, and PPG signals within a single wearable device. Analyses of individual and fused modalities enabled the extraction of key physiological features: PEP from ECG–PCG fusion, reflecting cardiac electromechanical function; PTT from PCG–PPG fusion, characterizing arterial stiffness and peripheral pulse propagation; and PAT from ECG–PPG fusion, capturing the integrated electromechanical coupling between the heart and arterial system.

PEP, an indicator of myocardial electromechanical coupling, was influenced by BMI but showed no association with age, suggesting that age-related cardiovascular changes primarily affect arterial rather than myocardial function. In contrast, PTT and the closely related PAT showed moderate negative correlations with both age and BMI, consistent with evidence that arterial stiffness increases with aging and excess body mass. These results highlight PTT as a potential non-invasive biomarker of vascular aging and cardiovascular risk. Moreover, PAT, which reflects combined heart–artery coupling, showed sex-based differences that warrant confirmation in larger, more diverse cohorts. Similarly, BMI-related findings are considered preliminary, given the narrow age and BMI ranges in this sample (mean age 29±7 years; BMI 22±3${\mathrm{kg}/m}^{2}$).

In conclusion, the proposed platform was intentionally developed using a wireless, low-power microcontroller that enables real-time data acquisition, onboard preprocessing, and wireless transmission. These capabilities support its integration into wearable or ambulatory monitoring systems. Future work will focus on implementing real-time feature extraction and cloud-based data streaming to facilitate continuous cardiovascular monitoring, early detection of hemodynamic changes, and personalized health tracking in both clinical and home environments. Nonetheless, the current study’s small and homogeneous sample (*n* = 14 healthy adults, aged 20–44) limits the generalizability and statistical power of the findings. Validation in larger and more diverse populations, including comparisons of PEP, PTT, and PAT with clinical standards such as echocardiography and arterial tonometry, is planned for future research.

## Data Availability

Data used and analyzed in this study can be obtained from the corresponding author upon reasonable request.
